# Prostaglandin Reductase 1 as a Potential Therapeutic Target for Cancer Therapy

**DOI:** 10.3389/fphar.2021.717730

**Published:** 2021-08-06

**Authors:** Xing Wang, Guobing Yin, Wei Zhang, Kunling Song, Longbin Zhang, Zufeng Guo

**Affiliations:** ^1^ Department of Breast and Thyroid Surgery, Second Affiliated Hospital of Chongqing Medical University, Chongqing, China; ^2^ Center for Novel Target and Therapeutic Intervention, Institute of Life Sciences, Chongqing Medical University, Chongqing, China

**Keywords:** PTGR1, tumor metabolism, eicosanoid pathways, cancer therapeutics, inhibitor

## Abstract

Altered tumor metabolism is a hallmark of cancer and targeting tumor metabolism has been considered as an attractive strategy for cancer therapy. Prostaglandin Reductase 1 (PTGR1) is a rate-limiting enzyme involved in the arachidonic acid metabolism pathway and mainly responsible for the deactivation of some eicosanoids, including prostaglandins and leukotriene B4. A growing evidence suggested that PTGR1 plays a significant role in cancer and has emerged as a novel target for cancer therapeutics. In this review, we summarize the progress made in recent years toward the understanding of PTGR1 function and structure, highlight the roles of PTGR1 in cancer, and describe potential inhibitors of PTGR1. Finally, we provide some thoughts on future directions that might facilitate the PTGR1 research and therapeutics development.

## Introduction

Cancer is a major global burden of disease and the second leading cause of death over a period of decades (Siegel et al., 2021; Sung et al., 2021). In recent years, cancer treatment and diagnosis have made remarkable progress, especially in immunotherapy (Hiam-Galvez et al., 2021; Salas-Benito et al., 2021), targeted therapies (Oh and Bang, 2020), molecular diagnostics (Sokolenko and Imyanitov, 2018), screening and early detection (Loud and Murphy, 2017) and so on. Although many cancers have become the chronic and even curable disease, tumor heterogeneity and acquired drug resistance remain two of the biggest challenges for cancer therapy, highlighting needs of novel anti-cancer strategies. Recently, an increasingly large body of evidence indicates that metabolic reprogramming, recognized as one of the cancer hallmarks (Hanahan and Weinberg, 2011), plays an essential role in cancer and targeting metabolic reprogramming has ability to develop novel strategies for cancer therapy (Fendt et al., 2020).

**FIGURE 1:**
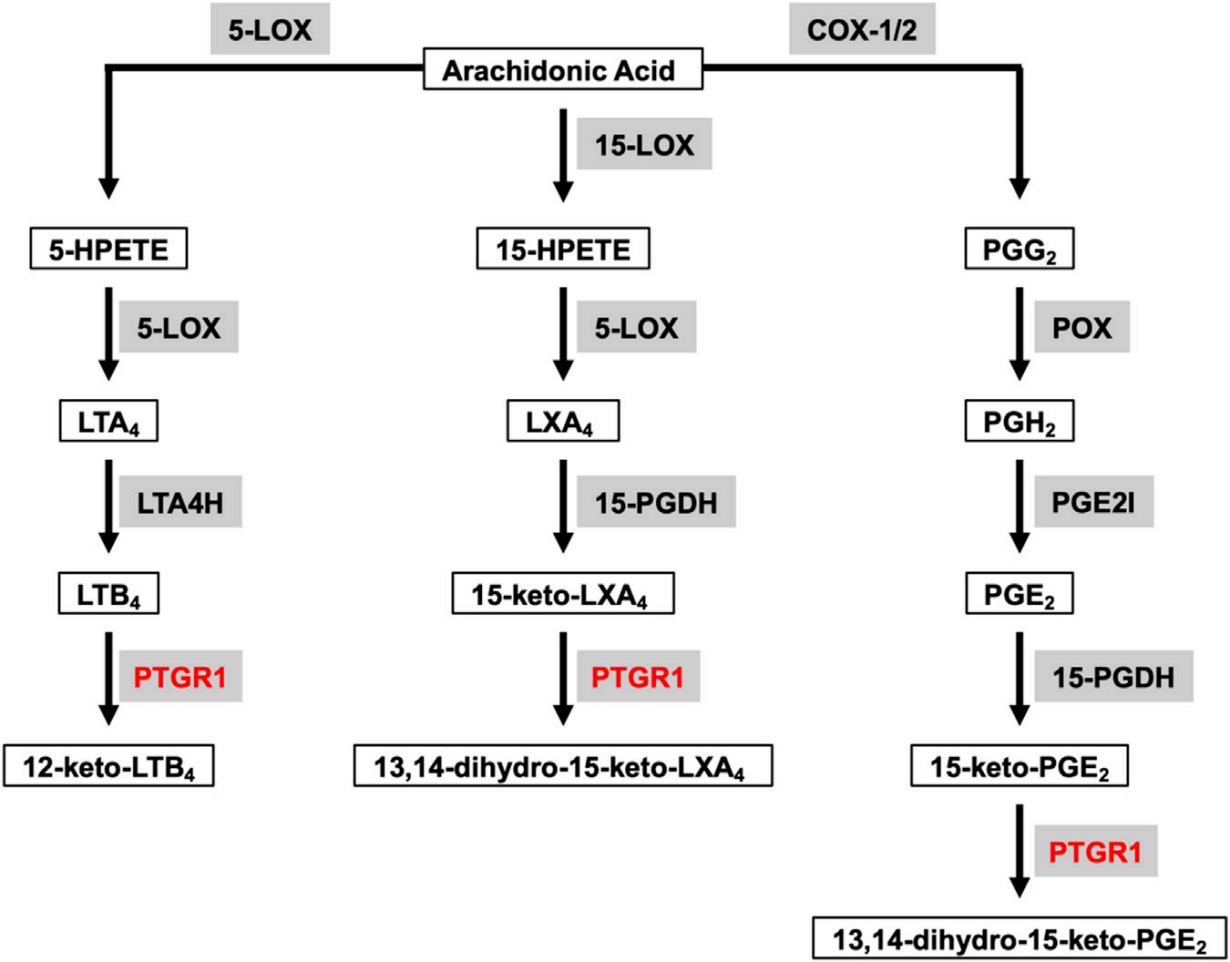
**Overview of PTGR1 function and its endogenous substrates.** Arachidonic Acid can be metabolized through COX and LOX pathways to generate eicosanoids, such as prostaglandins, leukotrienes and lipoxins. LTB_4_, 15-keto-LXA_4_, and 15-keto-PGE_2_ are the direct substrates for PTGR1. Substrates are emphasized with black box. Enzymes are emphasized in grey background and PTGR1 are colored with red. 5-LOX, 5-lipoxygenase; 15-LOX, 15-lipoxygenase; COX-1, cyclooxygenase-1; COX-2, cyclooxygenase-2; 5-HPETE, 5-hydroperoxyeicosatetraenoic acid; 15-HPETE, 15-hydroperoxyeicosatetraenoic acid; PGG_2_, PGH_2_, and PGE_2_ are prostaglandins G2, H2 and E2, respectively; LTA_4_ and LTB_4_ are leukotrienes A4 and B4, respectively; LXA_4_, lipoxin A4; LTA4H, leukotriene A4 hydrolase; 15-PGDH, 15-hydroxyprostaglandin dehydrogenase; PGE2I, prostaglandin E2 isomerase.

Arachidonic acid (AA) is a polyunsaturated fatty acid present in the phospholipids of cell membranes and can be metabolized through cyclooxygenase (COX), lipoxygenase (LOX) and P450 epoxygenase pathways to generate eicosanoids, including prostanoids (PGs), leukotrienes, hydroxy-eicosatetraenoic acids and so on (Wang et al., 2021). Prostaglandin Reductase 1 (PTGR1) belongs to the medium-chain dehydrogenase/reductase superfamily and it is involved in both COX and LOX downstream pathways to further metabolize eicosanoids (Figure 1). The altered metabolism of AA has long been shown to have crucial roles in cancer progression. For example, COX-2 expression is upregulated in multiple cancers, including colorectal, stomach, liver, lung and bladder, and increased COX-2 expression is associated with poor clinical outcomes. In cancer, the most abundant COX-2 product is prostaglandin E_2_ (PGE_2_) which has a predominant role in promoting tumor growth and is also associate with a poor prognosis (Wang and Dubois, 2010). LOX-derived leukotriene B4 (LTB4), through stimulating its receptor BLT1, has also been shown to promote TGF-β−mediated proliferation in breast cancer cells (Jeon et al., 2015).

Recently, a growing evidence suggested that PTGR1 plays a significant role in cancer progression (Xue et al., 2016), cancer prognosis (Győrffy et al., 2013), chemotherapeutic sensitivity (Dick et al., 2004) and cancer oxidative stress (Sánchez-Rodríguez et al., 2017). Therefore, PTGR1 is a novel potential therapeutical target for cancer treatment.

### The Function and Structure of PTGR1

PTGR1 is a NADPH-dependent alkenal/one oxidoreductase (AOR), which catalyzes the reduction of double bond of α/β-unsaturated ketones, alkenals and nitroalkenes. PTGR1 has broad spectrum of endogenous substrates and is involved in the degradation of PGs, LTB_4_, lipoxins and so on (Figure 1). LTB_4_ is one of the direct substrates of PTGR1 and can be oxidized to 12-keto-LTB_4_, which is approximately 100 times less potent than LTB_4_ (Yokomizo et al., 1993). However, PGs (such as PGE_2_, PGJ_2_, and PGF_2α_) are converted into 15-keto-PGs first by NAD-dependent 15-hydroxyprostaglandin dehydrogenase (15-PGDH), and then further metabolized into biologically less active 13,14-dihydro-15-keto-PGs by PTGR1. Similarly, LXA_4_ is oxidized to 15-keto-LXA_4_ by 15-PGDH and subsequently converted to 13,14-dihydro-15-keto-LXA_4_ by PTGR1 (Clish et al., 2000). In comparison to 15-keto-PGE_1_, 15-keto-PGF_1α_, 15-keto-PGF_2α_, and LTB_4_, 15-keto-PGE_2_ is the best substrate for PTGR1 with the highest *k*cat/*K*m value. For example, the *k*cat/*K*m value for 15-keto-PGE_2_ reduction is approximately 200 times higher than that for LTB_4_ oxidation (Mesa et al., 2015). Nitro oleic acid (NO-OA), another substrate of PTGR1, is an endogenous electrophilic compound with the capacity to modify thiols in proteins. PTGR1 is able to deactivate NO_2_-OA to non-electrophilic product NO_2_-SA through reduction (Vitturi et al., 2013). In addition to degradation of endogenous substrates, PTGR1 has ability to activate some drugs with α/β-unsaturated ketone. For example, CS-670 (Itoh et al., 2008), non-steroidal anti-inflammatory drugs (NSAIDs), and irofulven (hydroxymethylacylfulvene, HMAF) (McMorris et al., 1999) are converted into the active electrophilic metabolites by PTGR1 and then play their pharmacological role *in vitro* and *in vivo*.

There are only two structures of PTGR1 have been reported so far. In 2006, Hori et al. solved the crystal structure of guinea-pig PTGR1 in ternary complex with NADP^+^ and indomethacin and revealed that the broad spectrum of indomethacin efficacy can be attributed to its ability to adopt a range of different stable conformations with target proteins, including PTGR1 (Hori et al., 2006). In 2010, Yue et al. deposited the other unpublished structure of hPTGR1 in complex with NADP^+^ and raloxifene into Protein Data Bank (PDB) (Yue et al., 2011). Based on the hPTGR1 structure, Mesa et al. applied molecular docking simulations and site-directed mutagenesis assays to indicate that Arg56 plays an important role in binding the *α*-chain carboxylic group of 15-keto-PGE_2_ and 15‐d‐PGJ_2_ through an ionic interaction (Mesa et al., 2015). In addition, hPTGR1 inhibitors, niflumic acid and indomethacin have been found to establish ionic interactions with Arg56 through their carboxylic acid group, suggesting that the carboxylic acid stands as a good candidate pharmacophore group in the search of novel PTGR1 inhibitors (Mesa et al., 2015).

### PTGR1 and Cancer Progression

Overexpressed PTGR1 was found in many cancer cell lines, such as hepatocellular carcinoma (Sánchez-Rodríguez et al., 2017), lung cancer (Zhao et al., 2010), prostate cancer (Xue et al., 2016), and bladder cancer (Tapak et al., 2015), suggesting that PTGR1 has an oncogenic role. In addition, knockdown of PTGR1 was found to inhibit cell proliferation of multiple cancer cell lines. For example, Liu et al. found that knockdown of PTGR1 not only decreased the proportion of breast cancer stem cells, but also inhibited cell proliferation of TNBC cells (Liu et al., 2018). Similarly, Roberts et al. reported that knockdown of PTGR1 or inhibition of PTGR1 by its inhibitor was able to suppress TNBC cell growth (Roberts et al., 2017). In 2016, Xue et al. also demonstrated that knockdown of PTGR1 slowed down prostate cancer cell proliferation by inducing cell cycle arrest and apoptosis (Xue et al., 2016). In PC3 cells, PTGR1 silencing was found to increase the expression of key cell cycle inhibitor P21, cleaved-PARP and caspase 3, and decrease the expression of cyclin D1 (Xue et al., 2016). Together, these studies suggest that PTGR1 plays a role in cancer cell proliferation and can be of interest as a potential target for cancer therapy.

### PTGR1 and Cancer Prognosis

**FIGURE 2:**
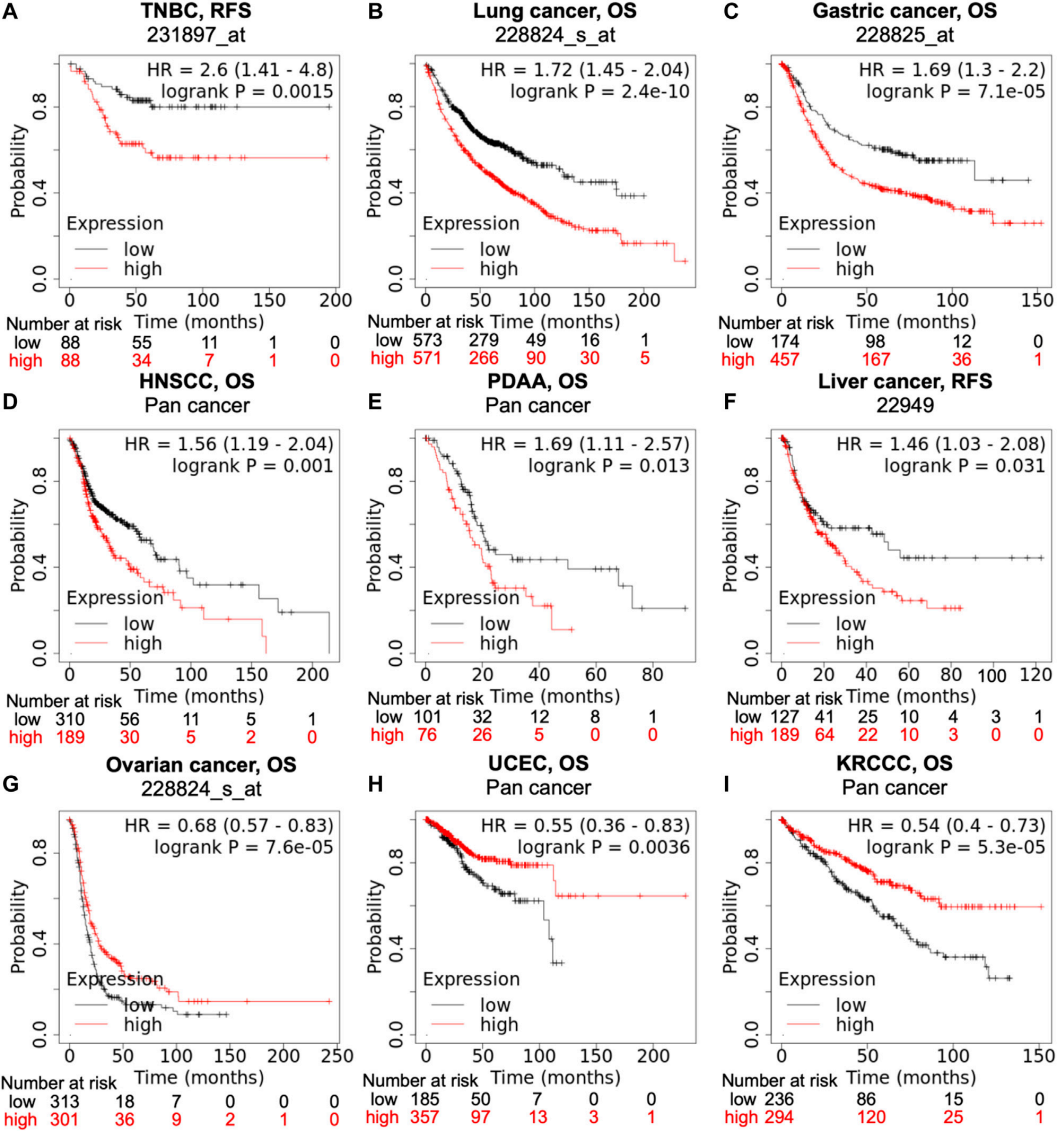
**Correlation of PTGR1 expression level with cancer prognosis.** Kaplan-Meier survival curves compare with the high and low expression of PTGR1 in different types of cancer in the Kaplan-Meier plotter **(A,B,C,F, and G)** and pan cancer databases **(D,E,H, and I)**. **(A–F)** High PTGR1 expression was correlated with poor RFS in TNBC and liver cancer, and poor OS in lung cancer, gastric cancer, HNSCC, and PDAA. **(G–I)** Low PTGR1 expression was correlated with poor OS in ovarian cancer, UCEC, and KRCC. Triple-negative breast cancer, TNBC; head-neck squamous cell carcinoma, HNSCC; pancreatic ductal adenocarcinoma, PDAA; uterine corpus endometrial carcinoma, UCEC; kidney renal clear cell carcinoma, KRCCC. OS, overall survival; RFS, relapse-free survival.

Data from the KM-plotter and Pan-cancer database suggests that high PTGR1 expression correlates with poor prognosis in a variety of tumor types, such as triple-negative breast cancer (TNBC), lung, gastric, head and neck, pancreatic and liver cancer (Figures 2A–F). On the contrary, in ovarian cancer, uterine corpus endometrial carcinoma, and kidney renal clear cell carcinoma, low PTGR1 level is related with the poor prognosis (Figures 2G–I). Recently, multiple studies also provided further evidence to support that the expression level of PTGR1 was related with cancer prognosis. First, Tapak et al. reported that the high expression of PTGR1 was associated with a decrease in survival time in bladder cancer (Tapak et al., 2015). Second, in colorectal cancer, Yang et al. found that low PTGR1 expression was associated with poor disease‐free survival in all stages (Yang et al., 2020). Last but not least, in gliomasphere model, Laks et al. demonstrated that low expression of PTGR1 was related to the aggressive phenotypes, including faster rate of proliferation, greater sphere total volume and increased sphere formation (Laks et al., 2016). These studies suggest that PTGR1 might be a novel prognostic biomarker in cancer and treatment with PTGR1 inhibitor or activator could benefit certain types of cancer patients.

### PTGR1 and Chemotherapeutic Sensitivity

As mentioned above, PTGR1 has ability to activate some prodrugs with α/β-unsaturated ketone, including anticancer agent HMAF (Raymond et al., 2004; Gong et al., 2006), suggesting that increasing PTGR1 expression level might enhance the efficacy of these drugs. Indeed, Dick et al. reported that human HEK293 cells with the overexpression of hPTGR1 were 100-times more sensitive than control cells to HMAF (Dick et al., 2004). They also confirmed that PTGR1 activity was positively correlated with HMAF sensitivity using NCI 60 human tumor cell line panel (Dick et al., 2004). In addition, PTGR1 expression was greatly enhanced by NRF2 inducers, such as D3T (Primiano et al., 1996), resveratrol (Yu et al., 2012) and curcumin (Yu et al., 2012). Yu et al. demonstrated that these NRF2 inducers significantly enhance the sensitivity of HMAF in colon and liver cancer cell lines from 2 to 10 fold (Yu et al., 2012). Therefore, these data indicate that PTGR1 could be used to design diagnostic tools or for combination cancer treatment.

### PTGR1 and Cancer Oxidative Stress

PTGR1 has alkenal/one oxidoreductase activity, suggesting that it might have an antioxidant effect against cancer oxidative stress. By studying PTGR1 expression during liver carcinogenesis, Sanchez-Rodriguez et al. found that PTGR1 stimulates cell proliferation and protected cells against ROS-induced cell death (Sánchez-Rodríguez et al., 2017). This result demonstrates that, indeed, PTGR1 has an antioxidant effect and perturbing the redox balance of tumor cells could be a strategy for inducing cell death. More recently, Hatem et al. discovered that auranofin (AUF) and vitamin C (VC) combinations show a synergistic and H_2_O_2_-mediated cytotoxicity to TNBC cell lines and higher PTGR1 expression are more resistance to AUF/VC combination (Hatem et al., 2019). These results suggest that PTGR1 could be used as an effective biomarker for response of cancer cells to ROS-mediated cancer treatment. In addition, PTGR1 inhibitor might sensitize cancer cells to ROS-induced cell death, which could be used to develop novel combination cancer treatment.

### PTGR1 Inhibitors

Although PTGR1 is a promising therapeutic target for cancer therapy, currently only a few PTGR1 inhibitors have been reported. NSAIDs are well-known inhibitors of COX-1 and COX-2 enzymes and able to reduce PTGR1 main substrate prostaglandins. Interestingly, some NSAIDs were also found to inhibit PTGR1. First, Clish et al. screened a panel of NSAIDs at 100 μM and found that indomethacin was the best PTGR1 inhibitor with more than 95% inhibition, while niflumic acid and diclofenac have about 80 and 70%, respectively, (Clish et al., 2001). Hori et al. also reported that indomethacin was able to inhibit the 15-keto-PGE_2_ reductase activity of guinea-pig PTGR1 with an IC_50_ of 97.9 µM (Hori et al., 2006). For hPTGR1, Mesa et al. demonstrated that niflumic acid and indomethacin were the best inhibitors with an IC_50_ of 7.1 and 8.7 µM, respectively, (Mesa et al., 2015). More recently, Roberts et al. reported that Licochalcone A blocked the NADP^+^ binding site of PTGR1 and inhibited cell growth in TNBC cells with an IC_50_ of 8.4 µM (Roberts et al., 2017).

### Discussion and Future Directions

In recent years, a growing body of literature has emphasized that metabolic enzyme PTGR1 plays a significant role in cancer and is a novel potential therapeutical target for cancer treatment. Although extensive efforts have been made, our current understanding of PTGR1 still remains at a juvenile stage.

First, no systematical approach was used to investigate the substrate spectrum of PTGR1. Metabolomic analysis on PTGR1 knockout cell lines may be useful to identify novel substates and provide the whole substrate spectrum for PTGR1. Second, most of the molecular understanding of PTGR1 is derived from experiments using docking simulations and site-directed mutagenesis assays. Solving of high-resolution 3D structures of hPTGR1 or complexed with its substrates and/or known inhibitors would further improve our knowledge of PTGR1’s function and help future computer-aided inhibitor design as well. Third, both PTGR1 upstream substrate PGE_2_ and direct substrate LTB_4_ play important roles in cancer progression through promoting tumor growth (Wang and Dubois, 2010; Jeon et al., 2015). This looks like in contrast with those studies demonstrating that knockdown of PTGR1 was able to inhibit cancer cell proliferation (Xue et al., 2016; Roberts et al., 2017; Liu et al., 2018). Currently limited information is available regarding the role of PTGR1 in cancer progression. One possibility is that PTGR1 might produce some metabolites, which play bigger pro-tumorigenic roles than PGE_2_ and LTB_4_ in multiple types of cancer cells, including at least TNBC and prostate cancer. Future studies should investigate the role of PTGR1 metabolites and focus on better understanding the inhibitory mechanisms of PTGR1 in cancer progression. Fourth, the correlation of PTGR1 and cancer survival remains questionable. Multiple studies have demonstrated that knockdown of PTGR1 can inhibit cell proliferation of TNBC cells (Roberts et al., 2017; Liu et al., 2018), which is consistent with the finding that high PTGR1 expression correlates with poor prognosis in TNBC cells. However, comparatively little is known why the correlation of PTGR1 and cancer survival exists in other cancer types and has opposing directions in different cancer types. It is worth noting that PTGR1 can change the sensitivity of some anticancer drugs, such as irofulven (McMorris et al., 1999). The correlation analysis will be affected if patients were treated with irofulven. Therefore, it is important to know the treatment status of the patients included in the analyses. Today, big data and AI are developing so fast, which is boosting the development of bioinformatic analysis. These methods would provide more evidence to answer whether PTGR1 is a novel biomarker for cancer prognosis. Finally, one of the most challenging yet exciting areas of future research will be the development of novel structure and high activity PTGR1 modulators, including inhibitors and activators. Currently, only a few PTGR1 inhibitors have been reported and none has entered clinical trials to date. All of them suffer from low activity with IC_50_ values at µM range. High-throughput screening, structure-based drug design, and virtual screening should be used to identify novel and potent PTGR1 modulators. These modulators will serve as chemical tools to further investigate the function of PTGR1 and drug leads for future cancer treatment. Interestingly, PTGR1 has also been linked to not only HMAF and other prodrug sensitivity, but also cancer oxidative stress. These results suggest that an PTGR1 activator might benefit HMAF anti-cancer treatment and an PTGR1 inhibitor might sensitize cancer cells to ROS-induced cell death. Therefore, combinational therapy approaches appear to be an interesting research direction for the utilization of PTGR1 modulators.
