# Corrigendum: Prostaglandin reductase 1 as a potential therapeutic target for cancer therapy

**DOI:** 10.3389/fphar.2023.1220144

**Published:** 2023-05-25

**Authors:** Xing Wang, Guobing Yin, Wei Zhang, Kunling Song, Longbin Zhang, Zufeng Guo

**Affiliations:** ^1^ Department of Breast and Thyroid Surgery, Second Affiliated Hospital of Chongqing Medical University, Chongqing, China; ^2^ Center for Novel Target and Therapeutic Intervention, Institute of Life Sciences, Chongqing Medical University, Chongqing, China

**Keywords:** PTGR1, tumor metabolism, eicosanoid pathways, cancer therapeutics, inhibitor

In the published article, an **author name** was incorrectly written as Kunlin Song. The correct spelling is Kunling Song.

The authors apologize for this error and state that this does not change the scientific conclusions of the article in any way. The original article has been updated.

